# Cellulosomal expansin: functionality and incorporation into the complex

**DOI:** 10.1186/s13068-016-0474-5

**Published:** 2016-03-12

**Authors:** Lior Artzi, Ely Morag, Melina Shamshoum, Edward A. Bayer

**Affiliations:** Department of Molecular Biosciences, The Weizmann Institute of Science, Rehovot, Israel

**Keywords:** Cohesin, Dockerin, Plant cell wall loosening, Cellulases, Cellulosomes, Tensile strength, Cellulose degradation, *Clostridium clariflavum*

## Abstract

**Background:**

Expansins are relatively small proteins that lack enzymatic activity and are found in plants and microorganisms. The function of these proteins is to disrupt the plant cell walls by interfering with the non-covalent interchain bonding of the polysaccharides. Expansins were found to be important for plant growth, but they are also expressed by various bacteria known to have interactions with plants. *Clostridium clariflavum* is a plant cell wall-degrading bacterium with a highly elaborate cellulosomal system. Among its numerous dockerin-containing genes, two expansin-like proteins, Clocl_1862 and Clocl_1298 (termed herein *Ccl*EXL1 and *Ccl*EXL2) were identified, and *Ccl*EXL1 was found to be expressed as part of the cellulosome system. This is the first time that an expansin-like protein is identified in a cellulosome complex, which implicates its possible role in biomass deconstruction.

**Results:**

In the present article, we analyzed the functionality of *Ccl*EXL1. Its dockerin was characterized and shown to bind selectively to type-I cohesins of *C. clariflavum*, with preferential binding to the cohesin of ScaG, and additionally to a type-I cohesin of *C. cellulolyticum*. We demonstrated experimentally that the expansin-like protein binds preferentially to microcrystalline cellulose, but it also binds to acid-swollen cellulose, xylan, and wheat straw. *Ccl*EXL1 exhibited a pronounced loosening effect on filter paper, which resulted in substantial decrease in tensile stress. The *C. clariflavum* expansin-like protein thus enhances significantly enzymatic hydrolysis of cellulose, both by *C. clariflavum* cellulosomes and two major cellulosomal cellulases from this bacterium: GH48 (exoglucanase) and GH9 (endoglucanase). Finally, we demonstrated *Ccl*EXL1-mediated enhancement of microcrystalline cellulose degradation by different cellulosome fractions and the two enzymes.

**Conclusions:**

The results of this study confirm that the *C. clariflavum* expansin-like protein is part of the elaborate cellulosome system of this bacterium with capabilities of cellulose creeping. The data suggest that pretreatment of cellulosic materials with *Ccl*EXL1 can bring about substantial improvement of hydrolysis by cellulases.

**Electronic supplementary material:**

The online version of this article (doi:10.1186/s13068-016-0474-5) contains supplementary material, which is available to authorized users.

## Background

As technologies develop and the standard of living continues to rise, energy costs and consequences are becoming too expensive and demanding for our dependence on unsustainable fossil fuel sources. Major efforts have been invested in recent years to find alternative fuel sources, and one of these is the use of lignocellulosic biomass for production of bioethanol [1–3]. This initiative has led to a growing interest in cellulolytic microorganisms and their carbohydrate-hydrolyzing systems as efficient resources for degrading cellulosic biomass.

Among the most efficient natural molecular machineries for cellulose degradation known today is the cellulosome [4]. The cellulosome is a multi-enzymatic complex that is produced by anaerobic cellulolytic bacteria and was first discovered in the thermophilic anaerobe *Clostridium thermocellum* [5–8]. Cellulosomes of different bacterial species are highly diverse and have different levels of complexity, depending on the “building blocks” that are encoded in the genome of a given cellulosome-producing species [5]. Cellulosomes are constructed of two major protein groups: structural subunits called “scaffoldins” and catalytic subunits, which are the enzymes [4, 9–12]. By definition, scaffoldins contain modules called “cohesins” that interact specifically with modules called “dockerins” that are found on the enzymatic subunits and/or other scaffoldins. A scaffoldin may also contain a carbohydrate-binding module (CBM) that targets the complex to the substrate [13–17]. The dockerin-containing proteins (notably the cellulosomal enzymes) integrate into the scaffoldin(s), thereby creating the mature cellulosome complex. Interactions between different scaffoldins can serve to multiply the number of enzymes in the complex and thus contribute to the complexity of these intriguing complexes [4]. The cohesin–dockerin complex is characterized by a strong, non-covalent, and highly specific protein–protein interaction, which exhibits inter- and/or intra-species specificity. There are different types of cohesins and dockerins, wherein the type-I cohesin–dockerin interaction is usually found between enzymes and scaffoldins, and the type-II cohesin–dockerin interaction usually occurs between different scaffoldins. The cohesin–dockerin interactions of each species are specific, and, in general, a cohesin/dockerin originating from one bacterial species cannot interact with a counterpart from another species, although there are exceptions [9, 18].

*Clostridium clariflavum* is a strict anaerobic, thermophilic, cellulolytic bacterium that was first reported in 2006 [19, 20]. Genome sequence analysis revealed that *C. clariflavum* contains a large number of cellulosomal genes [21]. In a previous study, we analyzed all of the cellulosomal genes of this species and identified 13 scaffoldins and 79 dockerin-containing proteins, which together create a highly elaborate and intricate cellulosome system. We defined the possible cohesin–dockerin interactions between the cellulosomal proteins (both bioinformatically and experimentally) [22], and the analysis revealed numerous cellulosome assemblies, the largest of which would contain up to 160 enzymatic subunits, which also represent the largest cellulosome complex known in nature today. Among the 79 dockerin-bearing proteins, we also identified two putative expansin-like proteins. This constitutes the first evidence for a cellulosomal expansin in nature.

Subsequently, we explored the status of this intricate cellulosomal system in vivo. As part of this effort, *C. clariflavum* was cultivated on three carbon sources, i.e., cellobiose (CB), microcrystalline cellulose (MCC), and switchgrass (SG) as sole carbon sources, and the cellulosome complexes were isolated from each of the spent growth media [23]. Cellulosome fractions were separated by size-exclusion chromatography in which two fractions for each growth medium were obtained: a high-molecular weight cellulosome fraction (CBI, MCCI, and SGI) and low-molecular-weight fraction (CBII, MCCII, and SGII). Cellulosome complexes with the highest catalytic activity were those isolated from the microcrystalline cellulose medium, i.e., MCCI and MCCII. We analyzed the protein contents of each fraction by quantitative LC–MS/MS and identified the key components of the different cellulosomes in each fraction. One of the interesting proteins that we identified during the analysis was the putative cellulosomal expansin-like protein Clocl_1862. This protein was only found in the low-molecular-weight complexes (CBII, MCCII, and SGII), and it was produced at relatively low expression levels—2 %, compared to the primary scaffoldin ScaA. Two putative cellulosomal expansin-like genes, Clocl_1862 (termed *Ccl*EXL1 for the purposes of this communication) and Clocl_1298 (*Ccl*EXL2), were identified in *C. clariflavum* genome. However, under the growth conditions applied in this study, only *Ccl*EXL1 was expressed by the bacterium; *Ccl*EXL2 was not detected.

Expansins are small proteins (~26 kDa) that lack catalytic activity and were first discovered in plants. These proteins were reported to have disruptive activity on plant cell walls and thereby assist growth and developmental processes in the plant [24–27]. The expansins are constructed of two small domains (D1 and D2) that are tightly associated to each other [27, 28]. Domain D1 exhibits some similarity to GH45 enzymes but lacks key residues that are critical for catalytic activity. Domain D2 was originally classified as CBM63 and is responsible mainly for substrate-binding characteristics, although it might have additional roles [28]. Although the expansins lack catalytic activity, they have the unique functionality of plant cell wall “creeping,” i.e., they interfere with non-covalent bonding of the plant cell wall polysaccharides and thus loosen the crystallinity of the substrate by physically separating associated polysaccharide chains [24].

Growing numbers of expansins have been discovered, both in plants and in bacterial species. Bacterial expansins have been found in species that are known to interact with plants, such as plant pathogens or bacteria that naturally inhabit plants [29]. Heterologous expression of plant expansin in *Escherichia coli* host expression systems results in very low and inefficient expression levels. However, expression of bacterial expansins generally results in high protein yields [26], which render these expansins of high interest for the field of biomass degradation studies and applications [30].

In recent years, many studies have attempted to demonstrate synergism between expansins and cellulases, but the effect of the expansins on cellulose hydrolysis remains debatable and inconclusive. Some of the studies have indeed reported synergistic effects of expansin and cellulases. These include the *Bs*EXLX1 expansin-like protein from *Bacillus subtilis* [31] and other bacterial species [32, 33] and the protein Zea h from corn stover [34]. In addition, the two *C. clariflavum* expansin-like proteins were previously integrated into small designer cellulosomes and added to the cellulose-binding fraction of the *C. clariflavum* and *C. thermocellum* secretomes [35]. However, other studies failed to find a synergistic effect between expansin-like proteins and cellulases [36], and even contradicted previous reports for the same proteins. In this context, Georgelis et al, failed to find a synergistic effect of *Bs*EXLX1 with several cellulases [29].

In the current study, we explored the functional characteristics of the *C. clariflavum* expansin-like protein *Ccl*EXL1, by experimentally addressing its cellulose-disruption activity, substrate-binding characteristics, the binding specificity of its dockerin module, and the impact of the expansin on the degradation of cellulosic substrates by *C. clariflavum* enzymes and cellulosomes. The information generated during the present study assists in understanding the role of a cellulosomal expansin in the cellulosome complex *per se* and suggests that this expansin can be used in different systems as an enhancer of the cellulose-hydrolysis process.

## Methods

### Cloning of *C. clariflavum* genes

*C. clariflavum* strain DSM 19,732 and its genomic DNA were purchased from the Leibniz Institute DSMZ-German Collection of Microorganisms and Cell Cultures. The genome sequence of *C. clariflavum* DSM 19,732 [CP003065.1] was obtained from the GenBank of NCBI. The genes encoding for the expansin-like protein *Ccl*EXL1 (Clocl_1862), GH9 (Clocl_2225), and GH48 (Clocl_4007) enzymes were cloned using specific primers for each gene: Expansin—forward primer: 5′-TATAACCATGGGCGAAGGACAAGTTACCGTTGGAGATATCAATGG-3′; Reverse primer: 5′-ATAATCTCGAGTTCAGGGAACTGGACATTGCCATCAATGATATAAGC-3′. GH9—forward primer: 5′-ATTATACCATGGGCCACCATCACCATCACCATGCAGGAAATTTCAACTATGC-3′; Reverse primer: 5′-TTATATCTCGAGTTAGAATGATTTTATTATTCC-3′. GH48—forward primer: 5′-TTATAACCATGGGCCACCATCACCATCACCATGACTCTGACACTTTTAAAGAC-3′; Reverse primer: 5′-TATATTCTCGAGTTAAAAATCTTTGCTTATTCCG-3′. All genes were designed to contain a His-tag, to facilitate purification of the recombinant proteins. The genes were amplified by polymerase chain reaction (PCR) using Reddymix × 2 (Advanced Biotechnologies Ltd., Epsom, Surrey, United Kingdom). The PCR products were purified by The HiYield gel-PCR fragment extraction kit (Real Biotech Corporation, RBC, Banqiao City, Taiwan). The inserts were restricted by NcoI (5′ terminus) and XhoI (3′ terminus) FastDigest enzymes (Thermo scientific, Fermentas UAB, Vilnius, Lithuania) and cloned into a pET28a plasmid using the NcoI and XhoI restriction sites. The plasmids were transformed to *E. coli* XL-1 Blue and purified by QIAprep Spin Miniprep Kit (QIAGEN GmbH, D-40724 Hilden, Germany). The recombinant scaffoldins Scaf·A, Scaf·B, Scaf·F, Scaf·G, Scaf·C, Scaf·T, CBM-CohA1, CBM-CohA5, CBM-CohA8, CBM-CohD3, and CBM-CohG were cloned as reported earlier [22, 37–40]. The trivalent Scaf·ABT was cloned as described previously by Vazana et al. [41].

### Protein expression and purification

The pET28a plasmids containing the recombinant genes were transformed to *E. coli* BL21 (DE3) cells. The cells were grown in LB (Luria–Bertani broth) medium supplemented with 50 µg/mL kanamycin (Sigma-Aldrich, Rehovot, Israel) and 2 mM CaCl_2_ to A_600_ ≈ 0.8 for 2.5 h at 37 °C. Protein expression was induced by the addition of 0.2 mM Isopropyl-1-thio-β-D-galactoside (IPTG) (Fermentas UAB, Vilnius, Lithuania). Following induction, cells were grown at 16 °C for 16 h. The cells were centrifuged at 5000 rpm for 15 min, and resuspended with TBS (Tris-buffered saline, 137 mM NaCl, 2.7 mM KCl, 25 mM Tris–HCl, pH = 7.4), supplemented with 5 mM imidazole. Cells were sonicated and centrifuged for 30 min at 15,000 rpm, 4 °C. Purification of the three proteins was performed in a batch purification system as described previously by Vazana et al. [42]. Protein purity was estimated by SDS-PAGE, and the proteins were dialyzed against TBS, supplemented with 5 mM CaCl_2_. The expression and purification of the recombinant Scaffoldins Scaf·A, Scaf·B, Scaf·F, Scaf·G, Scaf·C, Scaf·T, CBM-CohD3, CBM-CohA1, CBM-CohA5, CBM-CohA8, and CBM-CohG were carried out as described earlier [22, 37–40]. The expression and purification of Scaf·ABT was performed as reported before by Vazana et al. [41].

### Cultivation of *C. clariflavum* and isolation of its cellulosomes

*Clostridium clariflavum* was grown on GS-2 medium containing 0.2 % microcrystalline cellulose (Avicel; Sigma-Aldrich) as a sole carbon source [23]. The high-molecular-weight cellulosome fractions (MCCI—higher molecular weight fraction, MCCII—lower molecular weight fraction) were concentrated and purified as described previously [23].

### Affinity-based ELISA

The functionality of the N-terminal dockerin module of the *Ccl*EXL1 expansin was examined by affinity-based ELISA, based on the previous established protocol of Barak et al. [43]. Briefly, the 96-well ELISA plates (Greiner Bio-One, Belgium) were coated overnight with 30 nM of expansin. The five recombinant *C. clariflavum* scaffoldins (CBM-Cohs) CBM-CohA1, CBM-CohA5, CBM-CohA8, CBM-CohD3, and CBM-CohG, or 7 recombinant scaffoldins: Scaf·A, Scaf·B, Scaf·F, Scaf·G, Scaf·C, Scaf·T, and CBM-CohD3 were supplemented in concentrations ranging from 2 pM and 20 nM in order to evaluate their specific interaction profiles. The interactions were detected by anti-CBM primary antibody and horseradish peroxidase (HRP)-labeled secondary antibody.

### Affinity pulldown

In order to characterize the substrate-binding characteristics of *Ccl*EXL1, 10 µg of the expansin together with a negative control protein, Scaf·*ABT* [41], were incubated under continuous mixing for 1 h at 4 °C, in the presence of the following substrates: 11.25 mg microcrystalline cellulose (Avicel; Sigma-Aldrich), 5.6 mg/mL phosphoric acid-swollen cellulose, 11.25 mg beechwood xylan (Sigma-Aldrich, Rehovot, Israel), or 9 mg/mL of wheat straw (Valagro, Poitiers, France). As a control, a trivalent designer cellulosomal scaffoldin composed of three cohesin modules: *Acetivibrio cellulolyticus* cohesin (C3), *Bacteroides cellulosolvens* cohesin (B3), and *Clostridium thermocellum* cohesin (A2) [41] was tested under identical assay conditions. The reaction buffer was 50 mM acetate buffer, pH 5.5. Prior to addition of the *Ccl*EXL1, the beechwood xylan substrate was washed three times with double-distilled water, in order to remove free soluble saccharides. The final volume of the binding assay was 200 µL. After incubation, the tubes were centrifuged for 2 min at 14,000 rpm, the unbound fractions were collected and boiled in SDS sample buffer for 5 min at 100 °C. The pellets were washed three times with 200 µL of 50 mM acetate buffer (pH 5.5) with 0.05 % Tween 20 (Sigma-Aldrich, Rehovot, Israel), resuspended in reaction buffer and SDS sample buffer, boiled for 5 min at 100 °C, and centrifuged for 2 min, 14,000 rpm. The bound and unbound fractions were further analyzed by 12 % SDS-PAGE..

### Tensile-strength measurements of filter paper after different treatments

Expansins lack measurable enzymatic activity. Consequently, in order to determine whether this protein has biological functionality, we tested its influence on the tensile strength of filter paper after incubation in the presence of *Ccl*EXL1. The tensile strength of the samples was measured using an Instron 3345 Tester (Instron, Norwood, MA). The experiments were performed as described by Kim et al. [31]. Filter paper no. 3 strips at a size of 2 cm × 5.5 cm (Whatman, Little Chalfont, Buckinghamshire, UK) were incubated with 50 mM acetate buffer (pH 5.5), containing *Ccl*EXL1 (0.6 mg/mL). Bovine serum albumin (BSA, 0.6 mg/mL) or buffer alone was used as negative controls, and 8 M urea was used as a positive control. After 1 h of incubation at 55 °C in each solution, the tensile strength of the samples was measured. The extension rate used was 0.5 mm/min. Experiments were performed in triplicate, and standard deviations were determined.

### Hydrolysis of filter paper after incubation with expansin

Measurement of the tensile strength of filter paper after incubation with expansin showed that the filter paper weakens as a result of the expansin function. In order to demonstrate that the expansin disrupts the hydrogen bonds between the cellulose chains and makes it more accessible for the cellulases, we pre-incubated the filter paper strips with *Ccl*EXL1 (0.6 mg/mL in 50 mM acetate buffer, pH 5.5) for 1 h at 55 °C (or in the same buffer alone as a negative control). Subsequently, the recombinant enzymes GH48 and GH9 or the *C. clariflavum* cellulosomes (MCCI fraction) were added to the tubes. For comparison, the same reactions were performed without preincubation, i.e., the enzymes and the expansin were added simultaneously. The reactions were done in 200 µL volume, and the concentration of the enzymes was 0.5 µM each, and MCCI concentration was 25 µg/mL. The tubes were incubated for 24 h, at 55 °C, under continuous shaking. The amount of released reducing sugars was assessed by the DNS method [44], whereby enzymatic reactions were terminated by cooling the tubes on ice, 100 µL of the reaction volume was transferred into 150 µL of dinitrosalicylic acid (DNS), and the samples were boiled for 10 min at 100 °C. In addition, the residual filter paper samples were washed in double-distilled water, and the amount of the reducing sugars on the filter paper was also measured by the DNS method. After boiling, the samples were cooled on ice, and the absorbance was measured at 540 nm. The amount of reducing sugars was determined using a glucose standard curve. The experiments were performed three times in duplicates, the ± standard deviations for the three experiments are demonstrated.

### Enzymatic hydrolysis of Avicel by recombinant enzymes and expansin

Two prominent *C. clariflavum* cellulosome cellulases, GH48 and GH9, were used for enzymatic hydrolysis of Avicel, with or without the addition of *Ccl*EXL1. The addition of *Ccl*EXL1 was performed either simultaneously together with the enzymes or, alternatively, the microcrystalline cellulose samples were pre-incubated for 1 h at 55 °C prior to the supplementation of the enzymes. The reaction volume was 200 µL, and the enzymes and *Ccl*EXL1 were brought to a final concentration of 0.5 µM each. The tubes were supplemented with 11.25 mg of Avicel and were incubated at 55 °C for 24 h and 48 h with shaking. The amount of released reducing sugars was examined by the DNS method as described above.

### Hydrolysis of Avicel by *C. clariflavum* cellulosomes and expansin

In order to evaluate the contribution of expansin to cellulose degradation by the two cellulosome fractions, each fraction (MCCI and MCCII) was incubated with 11.25 mg of Avicel with or without *Ccl*EXL1. The concentration of each fraction was 25 µg/mL. The combination of the two fractions was also incubated with and without *Ccl*EXL1, and the total concentration of the two fractions was 25 µg/mL (12.5 µg/mL of each). *Ccl*EXL1 concentration was 0.5 µM. The tubes were incubated for 24, 48, and 72 h at 55 °C under continuous shaking. The levels of released reducing sugars were assessed as described above.

## Results

### Characterization of the binding specificity of the expansin-borne dockerin module

The *C. clariflavum* expansin-like gene Clocl_1862 encodes for a protein (*Ccl*EXL1) with a molecular mass of 35,122 Da, constructed of three domains: a dockerin module at the N-terminus of the protein and an expansin-like region, containing the two expansin-related domains (D1 and D2). The two expansin-like genes in the *C. clariflavum* genome are the first to be discovered to contain a type-I dockerin [22, 35], which renders them putative components of the cellulosome system in this bacterium. Two similar dockerin-bearing expansin-like proteins were also identified in the newly sequenced genome of *Clostridium alkalicellulosi* [45]. Moreover, the expansin-like protein *Ccl*EXL1 was also found to be part of cellulosome complexes that are produced by *C. clariflavum* when cultivated on different carbon sources [23]. Significantly, *Ccl*EXL1 was only identified in the lower-molecular-weight cellulosome fractions (the second fraction eluted by gel filtration during purification of *C. clariflavum* cellulosomes), and at relatively low expression levels (2 % relative to the amount of primary scaffoldin, ScaA, in this fraction). The second expansin-like protein, Clocl_1298 (*Ccl*EXL2), was not detected at all, upon cultivation of the bacterium on three different carbon sources (microcrystalline cellulose, cellobiose, and acid-pretreated switchgrass). Based on this observation, we decided to further characterize the *Ccl*EXL1 protein, which was expressed at very high levels in the *E. coli* expression system (yields of 70 mg/L were obtained), as reported for other highly expressed bacterial expansin-like proteins [26].

**Fig. 1:**
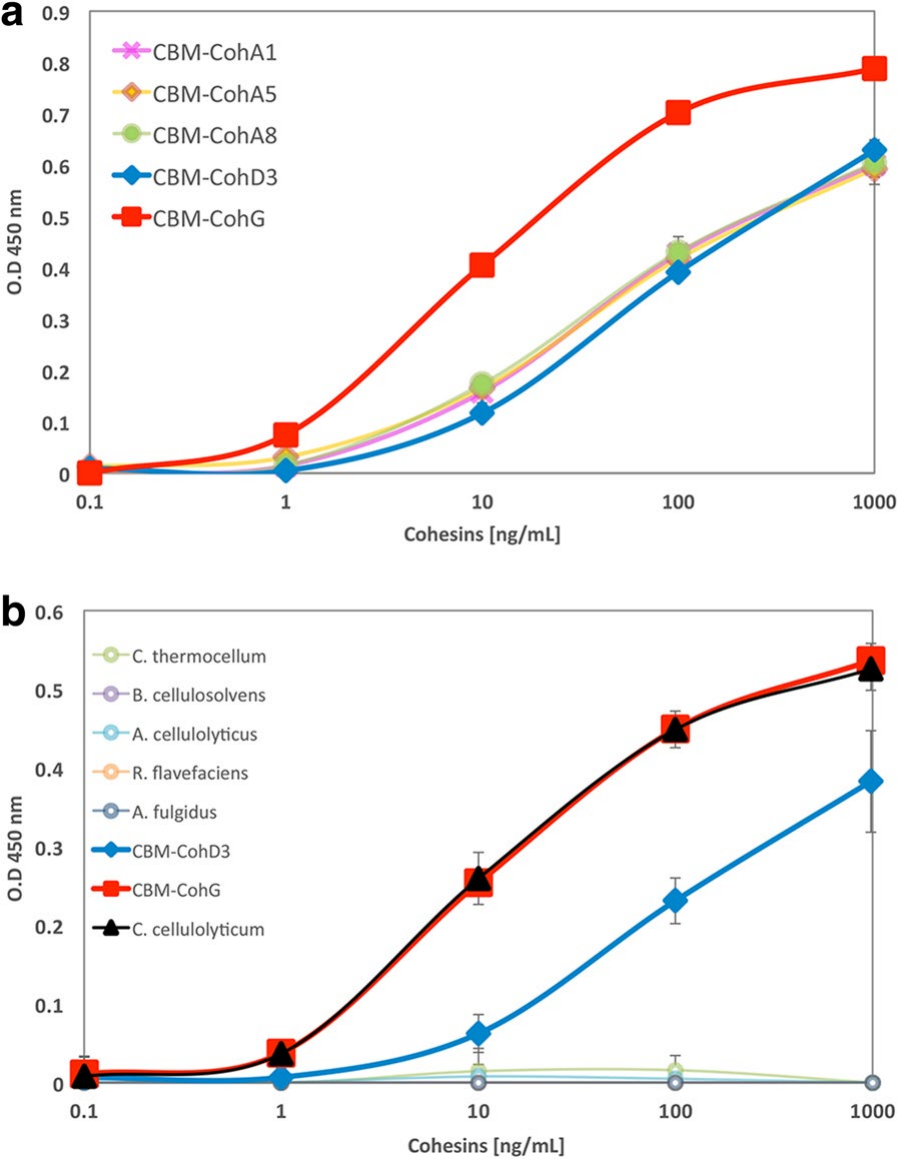
Investigation of the binding specificity of the expansin dockerin with various cohesins. The specific binding of the *Ccl*EXL1 dockerin was determined by affinity-based ELISA. Microtitter plates of 96 wells were coated with *Ccl*EXL1, and (**a**) five CBM-Coh fusion proteins from *C. clariflavum* (Cohesins A1, A5, A8, D3, and G) or (**b**) eight CBM-Coh fusion proteins from different bacterial species (*C. clariflavum*, *C. cellulolyticum*, *C. thermocellum*, *B. cellulosolvens*, *A. cellulolyticus*, *R. flavefaciens*, and *A. fulgidus*) were added to the wells in increasing concentrations. CBM, carbohydrate-binding module; Coh, cohesin

The binding characteristics of the type-I dockerin module of the expansin-like protein were examined by affinity-based ELISA. For this purpose, various monovalent recombinant scaffoldins (CBM-Cohs, each containing a CBM3 module and a single-cohesin module originating from a different bacterial species) were combined with *Ccl*EXL1. The expansin-like protein was found to interact with type-I cohesins from various scaffoldins of *C. clariflavum*, thereby confirming experimentally that this is a type-I dockerin. *Ccl*EXL1 interacts with the type-I cohesins of ScaA (CBM-Cohs A1, A5, and A8, the first, fifth, and eighth cohesins of ScaA), ScaD (CBM-CohD3, the third cohesin of ScaD), and the monovalent scaffoldin ScaG (CBM-CohG) [22] (Fig. 1a). The cohesins originating from ScaA and ScaD interacted at approximately the same level with the *Ccl*EXL1 dockerin module, whereas the ScaG cohesin interacted with its dockerin at a significantly higher level. For subsequent experiments, the two type-I cohesins from *C. clariflavum*, CBM-CohD3 and CBM-CohG, were chosen as positive controls for binding of the dockerin of *Ccl*EXL1. We then examined possible interactions of the latter with cohesins of other bacterial species. The other tested monovalent scaffoldins used in this work are as follows: Scaf·*A* (Cohesin C3 from *A. cellulolyticus*), Scaf·*B* (Cohesin B3 from *Bacteroides cellulosolvens*), Scaf·*F* (cohesin B1 from *Ruminococcus flavefaciens* strain 17), Scaf·*G* (cohesin 2375 from *Archaeoglobus fulgidus*), Scaf·*C* (cohesin C1 from *Clostridium cellulolyticum*), and Scaf·*T* (cohesin A from the CipA scaffoldin subunit of *Clostridium thermocellum* YS). All of the latter scaffoldins, with the exception of Scaf·*F*, have been established previously as type-I cohesins. As demonstrated in Fig. 1b, the dockerin of *Ccl*EXL1 did not interact with most of the monovalent scaffoldins, but exhibited specific intra-species interaction with CBM-CohD3, and again, a stronger binding to CBM-CohG. Surprisingly, however, *Ccl*EXL1 also displayed very strong and specific interspecies interaction the *C. cellulolyticum* cohesin [46, 47] at the same level as the intra-species interaction with the *C. clariflavum* CBM-CohG.

### Substrate-binding capabilities of *Ccl*EXL1

**Fig. 2:**
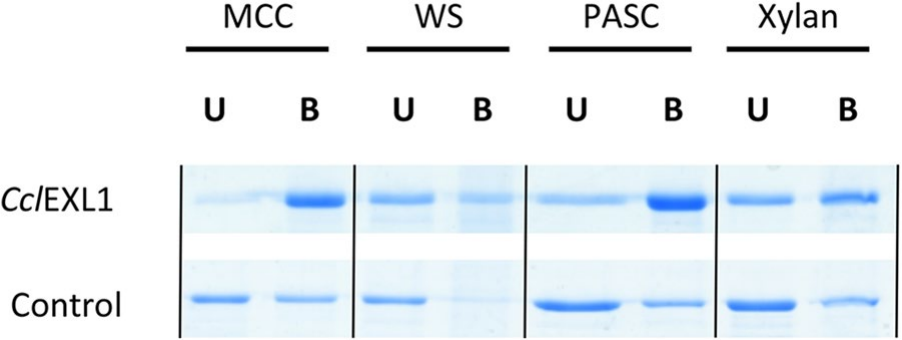
Affinity pulldown of *Ccl*EXL1 with cellulosic and hemicellulosic substrates. Microcrystalline cellulose (MCC), wheat straw (WS), phosphoric acid-swollen cellulose (PASC), and beechwood xylan were incubated with 10 µg of expansin for 1 h at 4 °C. The presence of *Ccl*EXL1 in the bound (B) and unbound (U) fractions was assessed by subjecting the fractions to 12 % SDS-PAGE. Scaf·ABT (10 µg) was used as a negative control

Expansins (both bacterial and of plant origin) are reported to contain two domains: D1 and D2, which together bind the substrate. D2 is the main binding component and is classified as a family-63 CBM. The bacterial expansin *Bs*EXLX1 from *B. subtilis* was shown to bind cellulose and different hemicelluloses [28]. For better understanding of the binding specificity of *Ccl*EXL1, we performed an affinity-pulldown assay with different substrates, i.e., microcrystalline cellulose, unpretreated wheat straw, phosphoric acid-swollen cellulose (PASC), and beechwood xylan. As a negative control, we used a trivalent recombinant scaffoldin Scaf·*ABT*, which lacks a CBM and comprises three cohesin modules, originating from *A. cellulolyticus*, *B. cellulosolvens,* and *C. thermocellum*. The results (Fig. 2) show that *Ccl*EXL1 binds strongly to cellulosic substrates, as most of the protein was found in the bound solid fraction (pellet) and only a very small portion remained in the unbound fraction (supernatant fluids). Interestingly, *Ccl*EXL1 bound microcrystalline cellulose in a stronger manner than the phosphoric acid-swollen cellulose, which might give a clue to the substrate preference of the expansin-like protein and to the available binding sites for expansin in each substrate. The control protein showed some presumably non-specific binding to the cellulosic substrates, but a larger portion of the protein was found in the unbound fraction.

In addition to cellulosic substrates, *Ccl*EXL1 also bound the hemicellulosic substrates—xylan and wheat straw. The binding to xylan was less extensive than the binding to cellulose, as can be seen by the amount of protein that remained in the unbound soluble fraction. The control protein also bound xylan in a non-specific manner, but again, a larger portion of the protein was found in the unbound soluble fraction. For wheat straw, only a very small portion of the control protein was found bound to the substrate, while most of the protein stayed in the unbound soluble fraction. This observation appears to reflect the binding preference of *Ccl*EXL1 to different plant cell wall polysaccharides, which favors binding to the cellulosic portion, although binding to hemicellulose was detected as well.

### The effect of the expansin-like protein on the tensile strength of filter paper

**Fig. 3:**
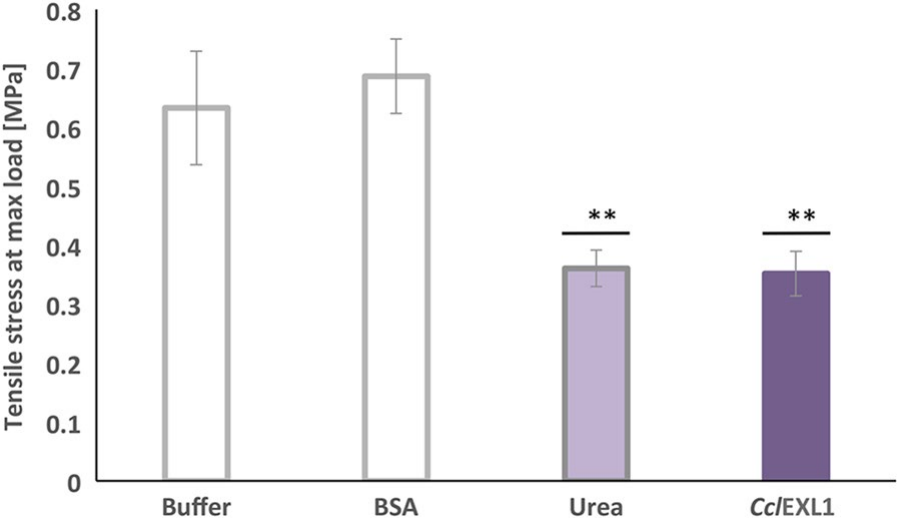
Examination of tensile strength of filter paper after different treatments. Filter paper strips were treated with acetate buffer (pH 5.5), 600 µg/mL BSA (as a negative control), 8 M urea (as a positive control), or 600 µg/mL *Ccl*EXL1 for 1 h at 55 °C, and the tensile strength of each strip was measured until tearing of the strip. *Standard deviations* are indicated, where *two* *asterisks* (**) indicate *p* < 0.01 (two-tailed *t* test for samples treated with buffer, urea, or *Ccl*EXL1)

Expansins lack detectable enzymatic activity. Although the D1 domain shares sequence and structural similarity with glycoside hydrolases from family 45, they lack selected key residues that are responsible for catalytic hydrolysis [26]. Previous studies have shown that expansins disrupt the non-covalent interactions in the plant cell wall and presumably interfere with the interchain hydrogen bonding of the crystalline cellulose structure, thus causing a “creeping” or “loosening” effect on the cell walls [24–26, 28]. In order to confirm that *Ccl*EXL1 exhibits the same biological function as other studied expansins, we measured the changes in tensile strength of filter paper as a result of 1-h incubation with *Ccl*EXL1. As negative controls, we incubated filter paper strips in acetate buffer (pH 5.5) alone or with the addition of BSA. This pH was chosen since this is the optimal pH for *C. clariflavum* cellulosome activity [23], and since *Ccl*EXL1 is an acidic expansin-like protein [48]. As a positive control, we incubated the strips in 8 M urea, which is known for its disruptive hydrogen bonding capacity and has commonly been used as a positive control by others. The results are shown in Fig. 3. Filter paper strips incubated with BSA were not affected and had similar values of tensile stress at maximum load as strips incubated in buffer. Strips incubated with 8 M urea exhibited a much lower value of tensile stress at maximum load, with a decrease of 43.0 % in tensile strength compared to strips incubated in buffer. The effect of *Ccl*EXL1 was almost identical to that of urea, with a decrease of 44.4 % in tensile strength compared to the controls. This level of filter paper weakening is significant, and the impact of *Ccl*EXL1 on tensile strength exceeded that of *Bs*EXLX1, which showed a 29 % decrease in tensile stress at maximum load (in experiments performed under identical conditions) [31]. These results provide direct experimental evidence that *Ccl*EXL1 possesses biological function consistent with expansins. It is also important to note that we examined whether *Ccl*EXL1 has catalytic activity on different substrates, but none was detected for any of the substrates.

### Enhancement of filter paper hydrolysis by cellulases as a result of preincubation with *Ccl*EXL1

**Fig. 4:**
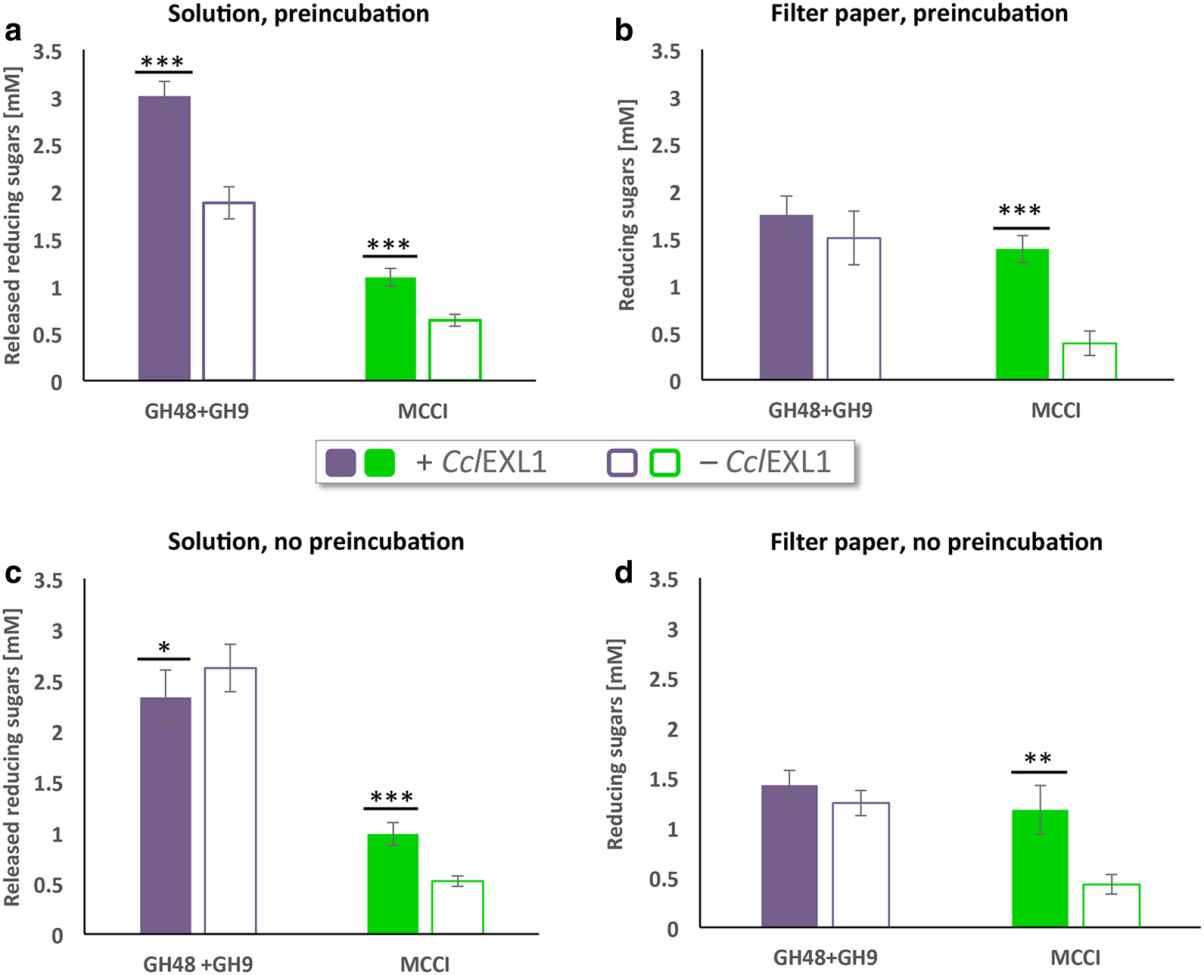
Preincubation of filter paper strips with *Ccl*EXL1 enhances enzymatic degradation. Filter paper strips were incubated for 1 h at 55 °C with either 600 µg/mL *Ccl*EXL1 or acetate buffer (pH 5.5) (**a**, **b**). For (**c**, **d**), no preincubation step was performed, and *Ccl*EXL1 was added to the reaction together with the enzymes and cellulosome. After incubation, the strips were supplemented with either 0.5 µM of two recombinant *C. clariflavum* cellulases, GH48 and GH9, or 25 µg/mL of the *C. clariflavum* MCCI cellulosome fraction, and hydrolysis was carried out for 24 h at 55 °C. The released reducing sugars in the solutions (**a**, **c**) and the reducing sugars on the filter paper (**b**, **d**) were measured. *Standard deviations* are indicated, where *three asterisks* (***) indicate *p* < 0.001, *two asterisks* (**) indicate *p* < 0.01, and a *single asterisk* indicates *p* < 0.05 (two-tailed *t* test)

Tensile-stress examination of filter paper after different treatments has shown that filter paper weakens as a result of preincubation with expansin. Our aim was thus to determine whether this effect can result in enhancement of cellulose hydrolysis by cellulases. For this purpose, we incubated filter paper strips with *Ccl*EXL1 or with acetate buffer for 1 h at 55 °C (as described for the tensile-stress measurements above). Immediately after incubation with *Ccl*EXL1, the filter paper was supplemented either by two purified cellulases or by the purified *C. clariflavum* cellulosomes (i.e., the high-molecular-weight fraction cellulosomes isolated from microcrystalline cellulose-grown cells, MCCI) for 24 h at 55 °C (Fig. 4a). As a control, the same experiment was performed with *Ccl*EXL1 but without preincubation, whereby CclEXL1 was added to the reaction at the same time with either the enzymes or with the cellulosome preparation (Fig. 4c, d). For this experiment, we cloned, expressed, and purified the putative *C. clariflavum* exoglucanase GH48-Doc (Clocl_4007) and the putative processive endoglucanase GH9-CBM3-Doc (Clocl_2225). Like in the majority of cellulosomes, the GH48 enzyme was found to be the most abundant enzyme in the cellulosome of *C. clariflavum*, and the GH9 enzyme is predicted to be a processive endoglucanase, similar to CelQ from *C. thermocellum*, and is also one of the highly expressed enzymes in the *C. clariflavum* cellulosome [23]. Examination of their catalytic activity on PASC showed that they work synergistically (Additional file 1). The samples of filter paper that were pretreated with *Ccl*EXL1 and hydrolyzed by either GH9 and GH48 or the MCCI cellulosome fraction showed a substantial increase in the level of the released reducing sugars in the reaction solution (59.71 or 71.14 %, respectively), compared to control samples that were incubated in buffer instead of *Ccl*EXL1 (Fig. 4a).

Examination of the reducing sugars found on the filter paper did not reflect the same increased hydrolysis effect by the GH48 and the GH9 enzymes, since the amounts of reducing sugars on the *Ccl*EXL1-treated filter paper and the non-treated filter paper were similar (Fig. 4b). However, filter paper samples that were hydrolyzed by the MCCI cellulosome preparation displayed a similar effect on the levels of reducing sugars as found in the reaction solution. These findings can be explained by the fact that the two enzymes have a lower capacity to hydrolyze and disrupt the structure of filter paper in comparison to the cellulosome, which contains a diversity of enzymes that work synergistically and together disrupt the filter paper more effectively. Preincubation with *Ccl*EXL1 assists both the enzymes and the cellulosomes in disrupting and hydrolyzing the filter paper, as shown in Fig. 4a. The high levels of released reducing sugars in the hydrolysis reaction with the two enzymes, compared to those remaining on the filter paper itself, can be explained by the fact that there is a high concentration of exoglucanase GH48 (compared to the amount of this enzyme in the MCCI fraction) that hydrolyzes the oligosaccharides released by endoglucanase GH9 to cellobiose subunits, which thus increases the levels of reducing ends in solution.

Filter paper samples that were not pre-incubated with *Ccl*EXL1 prior to the addition to the enzymes and cellulosome to the reaction (*Ccl*EXL1 was added together with the enzymes/cellulosome) revealed additional information (Fig. 4c, d). The levels of released reducing sugars in the solution as a result of the hydrolysis by the two enzymes was not increased by the addition of *Ccl*EXL1, and was even slightly reduced (Fig. 4c). However, hydrolysis of the filter paper by MCCI was enhanced by *Ccl*EXL1, independent of preincubation. An explanation for this phenomenon can be that the two enzymes and *Ccl*EXL1 compete for the same binding sites on the filter paper, and their simultaneous addition to the reaction prevents access of the enzymes to the filter paper. Upon preincubation of filter paper with *Ccl*EXL1, binding of *Ccl*EXL1 occurs earlier, and *Ccl*EXL1 can disrupt the filter paper and provide the enzymes with more exposed sites for degradation. When the cellulosome fraction is used instead of the two purified enzymes, the concentration of each enzyme in the complex is much lower, but a greater diversity of enzymes participate in hydrolysis of the filter paper. The enzymes work via different mechanisms and react on different sites of the filter paper, so in this case, *Ccl*EXL1 does not interfere with the hydrolysis, and hydrolysis is enhanced.

These observations reinforce the results of the tensile-strength measurements that show a weakening of the filter paper strips. These findings presumably reflect the disruption of hydrogen bonding between cellulose chains, which renders the substrate more accessible for enzymes, and cellulose hydrolysis is thus enhanced. No release of reducing sugars was detected for filter paper that was incubated under similar conditions with *Ccl*EXL1 alone without subsequent addition of enzymes, thus confirming its lack of catalytic activity.

### Catalytic hydrolysis of microcrystalline cellulose in the presence of *Ccl*EXL1

**Fig. 5:**
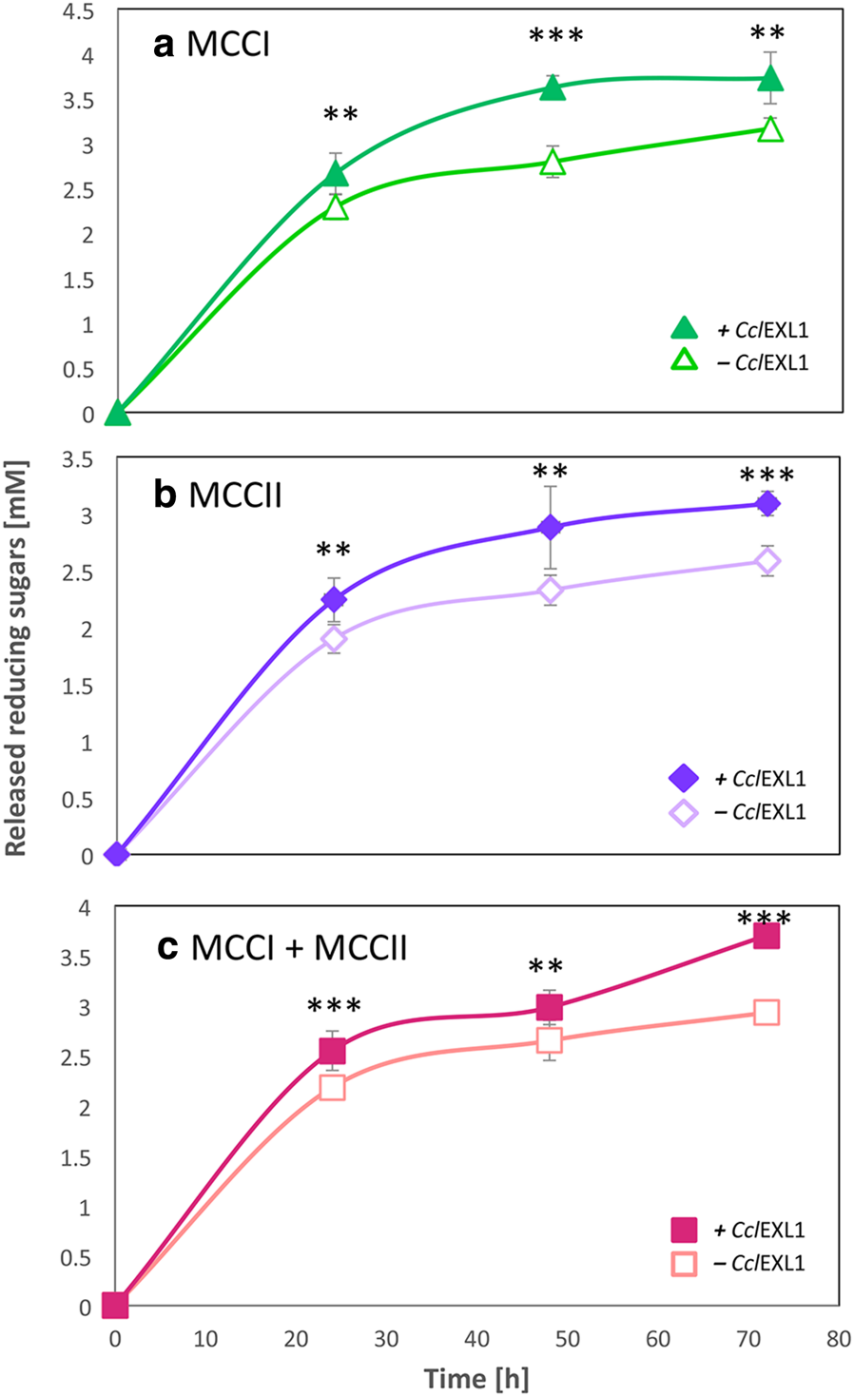
The impact of *Ccl*EXL1 on catalytic hydrolysis of microcrystalline cellulose by *C. clariflavum* cellulosome fractions. Hydrolysis of microcrystalline cellulose (*Avicel*) was performed by the cellulosome fractions of *C. clariflavum* (25 µg/mL) with (*full symbols*) or without (*empty symbols*) the addition of *Ccl*EXL1 (0.5 µM). Samples were taken at 24, 48, and 72 h, and the amount of the released reducing sugars was assessed. Hydrolysis was performed by the high-molecular-weight fraction, MCCI (**a**), the low-molecular-weight fraction, MCCII (**b**), and a combination of MCCI and MCCII (to a combined concentration of 25 µg/mL) (**c**). *Standard deviations* are indicated, where *two asterisks* indicate *p* < 0.01 and *three asterisks* indicate *p* < 0.001 (two-tailed *t* test)

The expansin of *C. clariflavum* contains a dockerin module at its N-terminus, which provides a clue about its role in nature. As a cellulosome component, *Ccl*EXL1 can be assumed to improve the activity of the cellulosomes in some way. We therefore isolated the cellulosomes of *C. clariflavum,* separated between the high-molecular-weight (MCCI) and the low-molecular-weight (MCCII) complexes by gel filtration (as described earlier [23]), and we examined whether addition of *Ccl*EXL1 would result in a synergistic effect on cellulose degradation (Fig. 5). For this purpose, *Ccl*EXL1 was added to each of the isolated cellulosome fractions (MCCI and MCCII) and to the combination of the two cellulosome complexes (MCCI + MCCII), and the effect on cellulose hydrolysis was determined. The hydrolysis of microcrystalline cellulose was performed at 55 °C, and was sampled at 24, 48, and 72 h. The concentration of each cellulosome fraction that was used was 25 µg/mL, and *Ccl*EXL1 was added to the reaction at a concentration of 0.5 µM. The combination of the MCCI and MCCII fractions was fixed at 25 µg/mL (12.5 µg/mL of each fraction). When *Ccl*EXL1 was the only protein in the reaction, no catalytic activity was observed. The results show that the addition of *Ccl*EXL1 to each of the cellulosome fractions causes a synergistic effect that fosters enhancement of cellulose hydrolysis. Catalytic hydrolysis of microcrystalline cellulose by MCCI in the presence of *Ccl*EXL1 was increased in comparison to the observed activity of MCCI alone (16.3, 29.7, and 17.7 % at 24, 48, and 72 h, respectively). For MCCII-mediated hydrolysis of cellulose, the observed activity was similarly enhanced (18, 23.7, and 19.5 % at the 24, 48, and 72 h, respectively) by *Ccl*EXL1, as observed also for the combined cellulosome fractions (MCCI + MCCII) (16.7, 12.5, and 26.3 % for 24, 48, and 72 h, respectively) (Fig. 5). These findings demonstrate that *Ccl*EXL1 has a significant synergistic effect on microcrystalline cellulose degradation, with both the high-molecular-weight cellulosomes and the low-molecular-weight cellulosome fractions. In addition, we performed a similar experiment in which *Ccl*EXL1 was added to the microcrystalline cellulose prior to the cellulosomes, in order to examine the influence of preincubation. The results showed that preincubation had no effect on microcrystalline cellulose hydrolysis, and the levels of enhancement by *Ccl*EXL1 remained at approximately ~20 % (data not shown).

However, when the concentration of the cellulosomes was increased (doubled) to 0.5 µg/mL, the synergistic effect of the expansin disappeared, and a negative synergistic effect was observed whereby *Ccl*EXL1 caused a minor decrease in the observed activity on microcrystalline cellulose (Additional file 2). A similar observation was reported earlier by Kim et al. 2009, whereby addition of the *B. subtilis* expansin-like protein BsEXEL1 caused an inhibition of filter paper hydrolysis by a commercial enzyme cocktail (Celluclast) comprising free uncomplexed fungal enzymes from *Trichoderma reesei* [31].

**Fig. 6:**
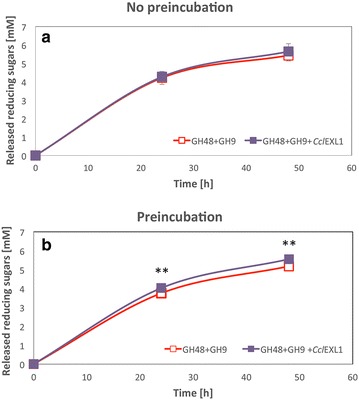
Hydrolysis of microcrystalline cellulose by two cellulases and *Ccl*EXL1. Degradation of Avicel was performed by exoglucanase GH48 and endoglucanase GH9 (0.5 µM each) with (*blue curve*) or without (*red curve*) the addition of *Ccl*EXL1 (0.5 µM). The reactions were performed with a simultaneous supplementation of enzymes and *Ccl*EXL1 (**a**) or with a 1-h preincubation step at 55 °C (**b**). *Two asterisks* indicate *p* < 0.01(two-tailed *t* test)

Finally, when the degradation of microcrystalline cellulose was performed by two cellulases, GH48 and GH9 and *Ccl*EXL1 (at an equimolar ratio of all components), the presence of expansin showed no substantial effect (Fig. 6a). This was surprising, since a potent synergistic effect was observed upon preincubation with *Ccl*EXL1 on this particular substrate prior to the action of these two cellulases (see above, Fig. 4). Furthermore, hydrolysis of microcrystalline cellulose was performed by the two enzymes after a preincubation period with *Ccl*EXL1, which resulted in 7.2 % enhancement of degradation (Fig. 6b). These results suggest that the synergistic effect of *Ccl*EXL1 depends on the composition of the enzymes in the reaction and/or their target substrates, and on preincubation of the substrates with *Ccl*EXL1 for different enzyme compositions.

## Discussion

Expansins are unique proteins that are expressed by plants and diverse types of bacteria, and their biological function has yet to be fully established. Various groups have shown in a number of studies that expansins from numerous sources are capable of plant cell wall “creeping” [24, 26, 31], but the exact mechanism of this activity is not known.

*C. clariflavum*, a cellulolytic bacterium with a highly elaborate cellulosomal system, contains in its genome two expansin-like genes, each bearing a dockerin module at their N-terminus. The dockerin module of the two expansin-like proteins is of type-I, as has been shown by sequence analysis of all non-scaffoldin dockerin-containing proteins of *C. clariflavum* [22], and we show herein that the dockerin module of expansin Clocl_1862 (*Ccl*EXL1) interacts with type-I cohesins of both *C. clariflavum* and *C. cellulolyticum* (Fig. 1). *C. clariflavum* has several different scaffoldins in its cellulosomal system that bear type-I cohesins: ScaA, which is the primary (enzyme-integrating) scaffoldin of the cellulosome and contains eight type-I cohesins; ScaC, an anchoring scaffoldin that bears three type-I cohesins, ScaD, another anchoring scaffoldin that comprises two type-II cohesins and a single (third) type-I cohesin; ScaG, which has a single type-I cohesin (also predicted to be a cell-surface anchoring scaffoldin); ScaH/L with three type-I cohesins; ScaM that also contains three type-I cohesins; ScaM (a) and ScaM(b), each bearing six type-I cohesins, and ScaO with a single type-I cohesin.

In our previous study [23] we performed quantitative label-free LC–MS/MS analysis of the *C. clariflavum* cellulosome fractions that revealed the complete protein contents of the cellulosomes. These data provided insight into the possible assemblies of the different components and the composition of dockerin-containing proteins in the cellulosome fractions [23]. *Ccl*EXL1 was found to be expressed by the bacterium, but at relatively low expression levels compared to other cellulosomal (enzyme) components. Intriguingly, this dockerin-bearing protein was found only in the low-molecular-weight fractions (CBII, MCCII, and SGII) for each cellulosome preparation isolated from cells grown on the three respective growth conditions. The amount of *Ccl*EXL1 in these fractions was only 2 % compared to that of the primary scaffoldin ScaA. *Ccl*EXL1 was not detected in the high-molecular-weight fractions (CBI, MCCI, and SGI), and the second expansin-like protein Clocl_1298 (*Ccl*EXL2) was not detected in any of the cellulosome fractions.

The low-molecular-weight cellulosome fractions of *C. clariflavum* contain three possible cellulosomal assemblies (Additional file 3). The first complex contains the primary scaffoldin ScaA that interacts with the anchoring scaffoldin ScaF through its single type-II cohesin. The second complex is composed of ScaM(b) that interacts with six dockerin-containing proteins, and the third is ScaG which contains a single type-I cohesin that interacts with a single dockerin-containing protein (see Fig. 3 of Ref. [23]). The fact that *Ccl*EXL1 is not found in the high-molecular-weight fractions can perhaps help deduce in which complex(es) it may likely reside. *Ccl*EXL1 probably does not interact with ScaA in the second fraction, since it is not found in the first (higher-molecular-weight) fraction where ScaA is dominant. Indeed, ScaA is the most abundant (or one of the most abundant) scaffoldin(s) in both the high- and low-molecular-weight fractions, so we may assume that if the expansin would have interacted with ScaA, we would also have found *Ccl*EXL1 in the high-molecular-weight fractions as well. If this is the case, the interaction of *Ccl*EXL1 would be restricted to ScaM(b) and/or ScaG. ScaM(b) is a cell-free scaffoldin, which is secreted from and would presumably diffuse away from the cell. An expansin that is incorporated into this scaffoldin would thus bind to the substrate via the scaffoldin-borne CBM2 and assist other enzymes in the complex to degrade the cellulosic substrate by disrupting the interchain hydrogen bonding, thereby rendering it more accessible to the enzymes. ScaG, on the other hand, is predicted to be a cell-anchored scaffoldin, and incorporation of *Ccl*EXL1 into this scaffoldin can result in the following possible roles: (i) adhesion of the cells to the substrate (probably cellulose), since expansin is composed of two domains, D1 and D2, and the latter mainly functions as a CBM; and (ii) enhancement of cellulose degradation by cell-anchored cellulosomes, assuming that cellulosomes are in close proximity to the *Ccl*EXL1-borne ScaG on the cell surface. These conclusions are supported by the affinity-based ELISA assay (Fig. 1), in which the cohesin of ScaG was shown to bind the dockerin of *Ccl*EXL1 stronger than the cohesins of ScaA and ScaD. However, an in vivo assay that will confirm preferential binding of *Ccl*EXL1 to ScaG and ScaM(b) is required.

The basic functionality of *Ccl*EXL1 was examined in our study employing two experimental approaches: the substrate-binding characteristics and the creeping activity on filter paper. The binding of cellulose by *Ccl*EXL1 was substantial, both to microcrystalline cellulose and PASC (Fig. 2). Surprisingly, *Ccl*EXL1 demonstrated more extensive binding to microcrystalline cellulose than to PASC, which is the reverse of the binding characteristics exhibited by many cellulases and/or CBMs. We observed that some of the *Ccl*EXL1 remained in the unbound fraction of the PASC-mediated pulldown experiment, even though the substrate was in great excess. It has been shown before that the binding of cellulases and CBM3 to PASC is 20-fold higher than to microcrystalline cellulose owing to the increased surface area of the former [15], which implies that the cellulases and CBM can access the cellulose more easily when neighboring cellulose chains are disrupted. These contrasting results may indicate that *Ccl*EXL1 can bind preferentially the crystalline regions of cellulose and work in such areas where expansin is required to facilitate cellulose hydrolysis. In addition, *Ccl*EXL1 was capable of binding partially xylan and wheat straw, which may suggest that it can also be active on hemicellulosic substrates.

Preincubation of filter paper with *Ccl*EXL1 resulted in significant reduction in tensile stress at maximum load of the filter paper (Fig. 3), which indicates that *Ccl*EXL1 possesses the same biological functionality as other characterized and well-studied expansins of different origins. This conclusion was further reinforced by the addition of cellulases or native cellulosome to the pre-incubated paper, which showed a substantial increase in the hydrolytic activity as a result of *Ccl*EXL1 action (Fig. 4). The preincubation step was effective only for degradation performed by the two enzymes, which indicated the importance of this step when working with artificial enzyme cocktails that might be inhibited by the expansin. Degradation of filter paper and microcrystalline cellulose by MCCI did not require this step, probably due to a much more complex enzyme composition. Similar results that show the importance of a preincubation step were reported by Tovar-Herrera et al. 2015 for ScExlx1, an expansin originating from *Schizophyllum commune* [49].

Moreover, examinations of tensile strength were performed under the same conditions reported by Kim et al. 2009 for *Bs*EXLX1 from *B. subtilis*, and the *C. clariflavum* expansin showed a markedly greater loosening effect on the filter paper (29 % for *Bs*EXLX1 versus 44.4 % for *Ccl*EXL1). Together with the fact that *Ccl*EXL1 is highly expressed in *E. coli* and is thermostable, these findings suggest that the *C. clariflavum* expansin *Ccl*EXL1 can be used as a biological pretreatment for cellulosic substrates, before adding the cellulases for enhanced substrate degradation.

Finally, we examined the effect of *Ccl*EXL1 on microcrystalline cellulose degradation by native *C. clariflavum* cellulosomes, separated into the high- and low-molecular-weight (MCCI and MCCII) fractions (Fig. 5). *Ccl*EXL1 enhanced cellulose hydrolysis by ~20 % for each of the cellulosome fractions and for its combination with MCCI and MCCII, but only when low concentrations of cellulosomes were employed. When a higher concentration was used, the synergistic effect diminished, and *Ccl*EXL1 even inhibited the degradation of the cellulosic substrate (Additional file 2). Similar findings have been reported in the literature [31]. A possible explanation of this phenomenon could be that *Ccl*EXL1 and the cellulosome compete for available binding sites on the cellulose surface, which causes an inhibition of enzyme action under high enzyme concentrations, since bound *Ccl*EXL1 blocks the binding of the enzymes to the substrate. Moreover, when *Ccl*EXL1 was added together with the two purified cellulases to microcrystalline cellulose, there was no detectable effect of the expansin on hydrolysis (Fig. 6), in contrast to its major contribution to the hydrolysis of filter paper, which was pre-incubated with expansin before addition of enzyme. However, when microcrystalline cellulose was pre-incubated with *Ccl*EXL1, a synergistic effect was observed. The results are compatible with those of Tovar-Herrera et al. 2015, who observed a synergistic effect of the expansin only when it was incubated with the substrate prior to enzymatic degradation. The improvement of the effect by preincubation with expansin might also be indicative of its location in the cellulosome system: if *Ccl*EXL1 would work independently of the enzymes, it might attach to the bacterial cell surface via ScaG and initiate substrate creeping before the cellulosome complex commences enzymatic hydrolysis. In any case, the precise role of *Ccl*EXL1 in the degradation of cellulosic substrates requires further experimental exploration.

These data suggest that the mechanism of *Ccl*EXL1 action is very complicated, and its contribution to biomass degradation depends on a variety of factors, such as the composition of enzymes used, the ratios between the expansin and the enzymes, the characteristics of the substrates that are used, and the timing of addition of the expansin to the substrate suspension. None of the studies that have been performed to date have succeeded in cracking the unique expansin code, but the evidence that has been collected from each study can eventually help in our efforts to better understand the mechanism of expansin action.

## Conclusions

The present study was initiated in order to address the role of the expansin-like protein, *Ccl*EXL1, in the *C. clariflavum* cellulosome system. We showed herein that *Ccl*EXL1 is capable of disrupting the substrate and assisting in cellulose degradation, both by recombinant enzymes and native cellulosomes. The *Ccl*EXL1 dockerin displayed preferential binding to the single cohesin of the presumed cell-anchored ScaG scaffoldin, thereby suggesting that its integration into the cellulosome system of this bacterium is not random. We demonstrated the importance of preincubation of the substrates with *Ccl*EXL1 when working with artificial enzyme cocktails, which can be highly beneficial to cellulolytic processes. Further studies are required for complete understanding of why this protein forms part of the enormous multi-enzymatic complex. It is currently a mystery why this bacterium produces an expansin-like protein and why other similar cellulosome-degrading bacteria, i.e*., C. thermocellum, A. cellulolyticus*, and *B. cellulosolvens,* do not. In the meantime, the secret remains with *C. clariflavum*.

## Additional files

10.1186/s13068-016-0474-5 Cellulases GH48 and GH9 work in a synergistic manner. The recombinant putative *C. clariflavum* exoglucanase GH48 and endoglucanase GH9 were used for degradation of PASC (phosphoric acid-swollen cellulose) alone or combined. Reaction tubes were supplemented with 0.5 µM of each enzyme, or 1 µM in total of the two enzymes combined. The duration of the reaction was 3 h, and the level of cellulose degradation was assessed by measuring the amount of released reducing sugars. The combination of the two enzymes resulted in 1.29-fold enhancement of PASC degradation. Synergy was calculated by summation of the released reducing sugars from the degradation by each enzyme alone, and comparing it to the amount of released reducing sugars by the action of the two enzymes together.
10.1186/s13068-016-0474-5 Impact of *Ccl*EXL1 is diminished at high cellulosome concentrations. Microcrystalline cellulose (Avicel) degradation was performed using the cellulosome fractions of *C. clariflavum,* MCCI (A), MCCII (B) and the combination of MCCI and MCCII (C), at a final concentration of 50 µg/mL with the addition of 0.5 µM expansin (full shapes) or without (empty shapes). Samples were taken at 24-h intervals, and the amount of released reducing sugars was assessed. The *Ccl*EXL1-mediated enhancement of cellulose hydrolysis that was demonstrated for low cellulosome concentrations (25 µg/mL) was not observed for higher concentrations. Standard deviations are indicated.
10.1186/s13068-016-0474-5 Low-molecular-weight cellulosomes of *C. clariflavum*. Expansin-like protein *Ccl*EXL1 was detected in the low-molecular-weight cellulosome fractions (CBII, MCCII and SGII) but not in the high-molecular-weight cellulosomes (Artzi et al. 2015). Based on tour previous data (Artzi et al. 2015) and the binding preferences of the CclEXL1 dockerin, the expansin may be a component of one or more of the following complexes: (i) Complex 1, *Ccl*EXL1 may interact with the type I cohesins of ScaA; (ii) Complex 2, *Ccl*EXL1 may interact with the ScaM(b) type I cohesins, or (iii) Complex 3, in which *Ccl*EXL1 may interact with the single cohesin of ScaG. Artzi L, Morag E, Barak Y, Lamed R, Bayer EA: *Clostridium clariflavum*: key cellulosome players are revealed by proteomic analysis. MBio 2015, 6:e00411–15.

## Abbreviations

BSA: bovine serum albumin
CBM: carbohydrate-binding module
Coh: cohesin
Doc: dockerin
Scaf: chimaeric scaffoldin
GH: glycoside hydrolase
PASC: phosphoric acid-swollen cellulose
MCC: microcrystalline cellulose
CB: cellobiose
SG: switchgrass
WS: wheat straw

## References

[1] Demain AL, Newcomb M, Wu JHD (2005). Cellulase, clostridia, and ethanol. Microbiol Mol Biol Rev.

[2] Ragauskas AJ, Williams CK, Davison BH, Britovsek G, Cairney J, Eckert CA, Frederick WJ, Hallett JP, Leak DJ, Liotta CL, Mielenz JR, Murphy R, Templer R, Tschaplinski T (2006). The path forward for biofuels and biomaterials. Science.

[3] Himmel ME, Ding SY, Johnson DK, Adney WS, Nimlos MR, Brady JW, Foust TD (2007). Biomass recalcitrance: engineering plants and enzymes for biofuels production. Science.

[4] Fontes CMGA, Gilbert HJ (2010). Cellulosomes: highly efficient nanomachines designed to deconstruct plant cell wall complex carbohydrates. Annu Rev Biochem.

[5] Bayer EA, Shoham Y, Lamed R, Rosenberg E (2013). Lignocellulose-decomposing bacteria and their enzyme systems. The Prokaryotes.

[6] Bayer EA, Kenig R, Lamed R (1983). Adherence of *Clostridium thermocellum* to cellulose. J Bacteriol.

[7] Lamed R, Setter E, Bayer EA (1983). Characterization of a cellulose-binding, cellulase-containing complex in *Clostridium thermocellum*. J Bacteriol.

[8] Lamed R, Setter E, Kenig R, Bayer EA (1983). The cellulosome—a discrete cell surface organelle of *Clostridium thermocellum* which exhibits separate antigenic, cellulose-binding and various cellulolytic activities. Biothecnology Bioeng Symp.

[9] Bayer EA, Morag E, Lamed R (1994). The cellulosome–a treasure-trove for biotechnology. Trends Biotechnol.

[10] Bayer EA, Chanzy H, Lamed R, Shoham Y (1998). Cellulose, cellulases and cellulosomes. Curr Opin Struct Biol.

[11] Bayer EA, Belaich J, Shoham Y, Lamed R (2004). The cellulosomes: multienzyme machines for degradation of plant cell wall polysaccharides. Annu Rev Microbiol.

[12] Doi RH, Kosugi A (2004). Cellulosomes: plant-cell-wall-degrading enzyme complexes. Nat Rev Microbiol.

[13] Shoseyov O, Takagi M, Goldstein MA, Doi RH (1992). Primary sequence analysis of *Clostridium cellulovorans* cellulose binding protein A. Proc Natl Acad Sci USA.

[14] Poole DM, Morag E, Lamed R, Bayer EA, Hazlewood GP, Gilbert HJ (1992). Identification of the cellulose-binding domain of the cellulosome subunit S1 from *Clostridium thermocellum* YS. FEMS Microbiol Lett.

[15] Morag E, Lapidot A, Govorko D, Lamed R, Wilchek M, Bayer EA, Shoham Y (1995). Expression, purification, and characterization of the cellulose-binding domain of the scaffoldin subunit from the cellulosome of *Clostridium thermocellum*. Appl Environ Microbiol.

[16] Shoseyov O, Shani Z, Levy I (2006). Carbohydrate binding modules: biochemical properties and novel applications. Microbiol Mol Biol Rev.

[17] Boraston AB, Bolam DN, Gilbert HJ, Davies GJ (2004). Carbohydrate-binding modules: fine-tuning polysaccharide recognition. Biochem J.

[18] Haimovitz R, Barak Y, Morag E, Voronov-Goldman M, Shoham Y, Lamed R, Bayer EA (2008). Cohesin-dockerin microarray: diverse specificities between two complementary families of interacting protein modules. Proteomics.

[19] Shiratori H, Ikeno H, Ayame S, Kataoka N, Miya A, Hosono K, Beppu T, Ueda K (2006). Isolation and characterization of a new *Clostridium sp.* that performs effective cellulosic waste digestion in a thermophilic methanogenic bioreactor. Appl Environ Microbiol.

[20] Shiratori H, Sasaya K, Ohiwa H, Ikeno H, Ayame S, Kataoka N, Miya A, Beppu T, Ueda K (2009). *Clostridium clariflavum* sp. nov. and *Clostridium caenicola* sp. nov., moderately thermophilic, cellulose-/cellobiose-digesting bacteria isolated from methanogenic sludge. Int J Syst Evol Microbiol.

[21] Izquierdo JA, Goodwin L, Davenport KW, Teshima H, Bruce D, Detter C, Tapia R, Han S, Land M, Hauser L, Jeffries CD, Han J, Pitluck S, Nolan M, Chen A, Huntemann M, Mavromatis K, Mikhailova N, Liolios K, Woyke T, Lynd LR (2012). Complete genome sequence of *Clostridium clariflavum* DSM 19732. Stand Genomic Sci.

[22] Artzi L, Dassa B, Borovok I, Shamshoum M, Lamed R, Bayer EA (2014). Cellulosomics of the cellulolytic thermophile *Clostridium clariflavum*. Biotechnol Biofuels.

[23] Artzi L, Morag E, Barak Y, Lamed R, Bayer EA (2015). *Clostridium clariflavum*: key cellulosome players are revealed by proteomic analysis. MBio.

[24] Sampedro J, Cosgrove DJ (2005). The expansin superfamily. Genome Biol.

[25] Cosgrove DJ (2005). Growth of the plant cell wall. Nat Rev Mol Cell Biol.

[26] Georgelis N, Nikolaidis N, Cosgrove DJ (2015). Bacterial expansins and related proteins from the world of microbes. Appl Microbiol Biotechnol.

[27] Yennawar NH, Li LC, Dudzinski DM, Tabuchi A, Cosgrove DJ (2006). Crystal structure and activities of EXPB1 (Zea m 1), a beta-expansin and group-1 pollen allergen from maize. Proc Natl Acad Sci USA.

[28] Georgelis N, Tabuchi A, Nikolaidis N, Cosgrove DJ (2011). Structure-function analysis of the bacterial expansin EXLX1. J Biol Chem.

[29] Georgelis N, Nikolaidis N, Cosgrove DJ (2014). Biochemical analysis of expansin-like proteins from microbes. Carbohydr Polym.

[30] Kim IJ, Lee HJ, Choi I, Kim KH (2014). Synergistic proteins for the enhanced enzymatic hydrolysis of cellulose by cellulase. Appl Microbiol Biotechnol.

[31] Kim ES, Lee HJ, Bang W-G, Choi I-G, Kim KH (2009). Functional characterization of a bacterial expansin from *Bacillus subtilis* for enhanced enzymatic hydrolysis of cellulose. Biotechnol Bioeng.

[32] Bunterngsook B, Eurwilaichitr L, Thamchaipenet A, Champreda V (2015). Binding characteristics and synergistic effects of bacterial expansins on cellulosic and hemicellulosic substrates. Bioresour Technol.

[33] Bunterngsook B, Mhuantong W, Champreda V, Thamchaiphenet A, Eurwilaichitr L (2014). Identification of novel bacterial expansins and their synergistic actions on cellulose degradation. Bioresour Technol.

[34] Han Y, Chen H (2007). Synergism between corn stover protein and cellulase. Enzyme Microb Technol.

[35] Chen C, Cui Z, Song X, Liu YJ, Cui Q, Feng Y (2016). Integration of bacterial expansin-like proteins into cellulosome promotes the cellulose degradation. Appl Microbiol Biotechnol.

[36] Olarte-Lozano M, Mendoza-Nuñez MA, Pastor N, Segovia L, Folch-Mallol J, Martínez-Anaya C (2014). PcExl1 a novel acid expansin-like protein from the plant pathogen *Pectobacterium carotovorum*, binds cell walls differently to BsEXLX1. PLoS One.

[37] Morais S, Barak Y, Caspi J, Hadar Y, Lamed R, Shoham Y, Wilson DB, Bayer EA (2010). Cellulase-xylanase synergy in designer cellulosomes for enhanced degradation of a complex cellulosic substrate. MBio.

[38] Moraïs S, Barak Y, Caspi J, Hadar Y, Lamed R, Shoham Y, Wilson DB, Bayer EA (2010). Contribution of a xylan-binding module to the degradation of a complex cellulosic substrate by designer cellulosomes. Appl Environ Microbiol.

[39] Morais S, Barak Y, Hadar Y, Wilson DB, Shoham Y, Lamed R, Bayer EA (2011). Assembly of xylanases into designer cellulosomes promotes efficient hydrolysis of the xylan component of a natural recalcitrant cellulosic substrate. MBio.

[40] Haimovitz R, Barak Y, Morag E, Voronov-Goldman M, Shoham Y, Lamed R, Bayer EA (2008). Cohesin-dockerin microarray: diverse specificities between two complementary families of interacting protein modules. Proteomics.

[41] Vazana Y, Barak Y, Unger T, Peleg Y, Shamshoum M, Ben-Yehezkel T, Mazor Y, Shapiro E, Lamed R, Bayer EA (2013). A synthetic biology approach for evaluating the functional contribution of designer cellulosome components to deconstruction of cellulosic substrates. Biotechnol Biofuels.

[42] Vazana Y, Moraïs S, Barak Y, Lamed R, Bayer EA (2010). Interplay between *Clostridium thermocellum* family 48 and family 9 cellulases in cellulosomal versus noncellulosomal states. Appl Environ Microbiol.

[43] Barak Y, Handelsman T, Nakar D, Mechaly A, Lamed R, Shoham Y, Bayer EA (2005). Matching fusion protein systems for affinity analysis of two interacting families of proteins: the cohesin-dockerin interaction. J Mol Recognit.

[44] Miller GL (1959). Use of dinitrosalicylic acid reagent for determination of reducing sugar. Anal Chem.

[45] Zavarzin GA, Zhilina TN, Dulov LE (2008). Alkaliphilic sulfidogenesis on cellulose by combined cultures. Microbiology.

[46] Pinheiro BA, Proctor MR, Martinez-Fleites C, Prates JAM, Money VA, Davies GJ, Bayer EA, Fontes CMGA, Fierobe HP, Gilbert HJ (2008). The *Clostridium cellulolyticum* dockerin displays a dual binding mode for its cohesin partner. J Biol Chem.

[47] Mechaly A, Fierobe HP, Belaich A, Belaich JP, Lamed R, Shoham Y, Bayer EA (2001). Cohesin-dockerin interaction in cellulosome assembly: a single hydroxyl group of a dockerin domain distinguishes between nonrecognition and high affinity recognition. J Biol Chem.

[48] Pastor N, Dávila S, Pérez-Rueda E, Segovia L, Martínez-Anaya C (2015). Electrostatic analysis of bacterial expansins. Proteins Struct Funct Bioinforma.

[49] Tovar-Herrera OE, Batista-García RA, Sánchez-Carbente MDR, Iracheta-Cárdenas MM, Arévalo-Niño K, Folch-Mallol JL (2015). A novel expansin protein from the white-rot fungus *Schizophyllum commune*. PLoS One.

