# Supplementary material for: Cellulosomal expansin: functionality and incorporation into the complex
Source: Biotechnol Biofuels. 2016 Mar 12;9:61. doi: 10.1186/s13068-016-0474-5 (PMC4788839; doi:10.1186/s13068-016-0474-5)
Supplement: Supplementary file 1 — 10.1186/s13068-016-0474-5 Cellulases GH48 and GH9 work in a synergistic manner. The recombinant putative C. clariflavum exoglucanase GH48 and endoglucanase GH9 were used for degradation of PASC (phosphoric acid-swollen cellulose) alone or combined. Reaction tubes were supplemented with 0.5 µM of each enzyme, or 1 µM in total of the two enzymes combined. The duration of the reaction was 3 h, and the level of cellulose degradation was assessed by measuring the amount of released reducing sugars. The combination of the two enzymes resulted in 1.29-fold enhancement of PASC degradation. Synergy was calculated by summation of the released reducing sugars from the degradation by each enzyme alone, and comparing it to the amount of released reducing sugars by the action of the two enzymes together. [file 13068_2016_474_MOESM1_ESM.docx]

**Figure S1.** **Cellulases GH48 and GH9 work in a synergistic manner.** The recombinant putative *C. clariflavum* exoglucanase GH48 and endoglucanase GH9 were used for degradation of PASC (phosphoric acid-swollen cellulose) alone or combined. Reaction tubes were supplemented with 0.5 µM of each enzyme, or 1 µM in total of the two enzymes combined. The duration of the reaction was 3 h, and the level of cellulose degradation was assessed by measuring the amount of released reducing sugars. The combination of the two enzymes resulted in 1.29-fold enhancement of PASC degradation. Synergy was calculated by summation of the released reducing sugars from the degradation by each enzyme alone, and comparing it to the amount of released reducing sugars by the action of the two enzymes together.
