# Supplementary material for: Cellulosomal expansin: functionality and incorporation into the complex
Source: Biotechnol Biofuels. 2016 Mar 12;9:61. doi: 10.1186/s13068-016-0474-5 (PMC4788839; doi:10.1186/s13068-016-0474-5)
Supplement: Supplementary file 2 — 10.1186/s13068-016-0474-5 Impact of CclEXL1 is diminished at high cellulosome concentrations. Microcrystalline cellulose (Avicel) degradation was performed using the cellulosome fractions of C. clariflavum, MCCI (A), MCCII (B) and the combination of MCCI and MCCII (C), at a final concentration of 50 µg/mL with the addition of 0.5 µM expansin (full shapes) or without (empty shapes). Samples were taken at 24-h intervals, and the amount of released reducing sugars was assessed. The CclEXL1-mediated enhancement of cellulose hydrolysis that was demonstrated for low cellulosome concentrations (25 µg/mL) was not observed for higher concentrations. Standard deviations are indicated. [file 13068_2016_474_MOESM2_ESM.docx]

**Figure S2**: Impact of *Ccl*EXL1 is diminished at high cellulosome concentrations. Microcrystalline cellulose (Avicel) degradation was performed using the cellulosome fractions of *C. clariflavum,* MCCI (A), MCCII (B) and the combination of MCCI and MCCII (C), at a final concentration of 50 µg/mL with the addition of 0.5 µM expansin (full shapes) or without (empty shapes). Samples were taken at 24-h intervals, and the amount of released reducing sugars was assessed. The *Ccl*EXL1-mediated enhancement of cellulose hydrolysis that was demonstrated for low cellulosome concentrations (25 µg/mL) was not observed for higher concentrations. Standard deviations are indicated.
