# Supplementary material for: Cellulosomal expansin: functionality and incorporation into the complex
Source: Biotechnol Biofuels. 2016 Mar 12;9:61. doi: 10.1186/s13068-016-0474-5 (PMC4788839; doi:10.1186/s13068-016-0474-5)
Supplement: Supplementary file 3 — 10.1186/s13068-016-0474-5 Low-molecular-weight cellulosomes of C. clariflavum. Expansin-like protein CclEXL1 was detected in the low-molecular-weight cellulosome fractions (CBII, MCCII and SGII) but not in the high-molecular-weight cellulosomes (Artzi et al. 2015). Based on tour previous data (Artzi et al. 2015) and the binding preferences of the CclEXL1 dockerin, the expansin may be a component of one or more of the following complexes: (i) Complex 1, CclEXL1 may interact with the type I cohesins of ScaA; (ii) Complex 2, CclEXL1 may interact with the ScaM(b) type I cohesins, or (iii) Complex 3, in which CclEXL1 may interact with the single cohesin of ScaG. Artzi L, Morag E, Barak Y, Lamed R, Bayer EA: Clostridium clariflavum: key cellulosome players are revealed by proteomic analysis. MBio 2015, 6:e00411–15. [file 13068_2016_474_MOESM3_ESM.docx]

**Figure S3**: **Low-molecular-weight cellulosomes of *C. clariflavum*.** Expansin-like protein *Ccl*EXL1 was detected in the low-molecular-weight cellulosome fractions (CBII, MCCII and SGII) but not in the high-molecular-weight cellulosomes (Artzi et al., 2015). Based on tour previous data (Artzi et al., 2015) and the binding preferences of the CclEXL1 dockerin, the expansin may be a component of one or more of the following complexes: (i) Complex 1, *Ccl*EXL1 may interact with the type I cohesins of ScaA; (ii) Complex 2, *Ccl*EXL1 may interact with the ScaM(b) type I cohesins, or (iii) Complex 3, in which *Ccl*EXL1 may interact with the single cohesin of ScaG.

Artzi L, Morag E, Barak Y, Lamed R, Bayer EA: ***Clostridium clariflavum*: key cellulosome players are revealed by proteomic analysis**. *MBio* 2015, **6**:e00411–15.
